# Improvement of the Quality of Life in Patients with Age-Related Macular Degeneration by Using Filters

**DOI:** 10.3390/ijerph17186751

**Published:** 2020-09-16

**Authors:** Daniel Caballe-Fontanet, Cristina Alvarez-Peregrina, Neus Busquet-Duran, Eduard Pedemonte-Sarrias, Miguel Angel Sanchez-Tena

**Affiliations:** 1 School of Biomedical and Health Science, Universidad Europea de Madrid, 28670 Madrid, Spain; opticavisiocaballe@gmail.com (D.C.-F.); cristina.alvarez@universidadeuropea.es (C.A.-P.); 2 Department of Ophthalmology, Althaia Xarxa Assistencial Universitària de Manresa, 08243 Manresa, Spain; nbusquet@althaia.cat (N.B.-D.); epedemonte@althaia.cat (E.P.-S.); 3 Central University of Catalonia (UVic-UCC), Faculty of Medicine, University of Vic, 08500 Vic, Spain

**Keywords:** age-related macular degeneration, NEI VFQ-25, selective cut optical filters, quality of life

## Abstract

Background: Age-related macular degeneration (AMD) is a disease with an increasing incidence due to the general aging of the population that decreases the patient’s quality of life. This work aims to study whether selective cut optical filters improve the AMD patient’s quality of life. Methods: Prospective and longitudinal study in 79 patients. Visual acuity, contrast sensitivity, and the line differences in the Colenbrander test were measured. Patients answered The National Eye Institute 25-Item Visual Function Questionnaire (NEI VFQ-25), which measures the quality of life related to vision before and after using cut optical filters. Results: There was an improvement of 5.99 points (3.7–8.3) in NEI VFQ-25 after wearing filters. This improvement was 4.0 points for 450-nm filters and 12.7 points for 511-nm filters. For patients with visual acuity (VA) < 0.25, results of NEI VFQ-25 increased by 10.11 points (1.19–19.02) and for patients with late AMDs, results increased by 5.33 points (1.31–9.35). Conclusions: Selective filters improve the quality of life of patients with AMD. The success rate in the fitting of filters is better for those with VA lower than 0.25 and those with late or advanced AMD.

## 1. Introduction

Age-related macular degeneration (AMD) is a disease with an increasing incidence due to the general aging of the population. It is the main cause of blindness in the USA and the third cause of blindness in the world [1]. Depending on the age, prevalence varies, ranging from 3.4% at 65 years of age to 12.2% at 80. There are no differences in sex, although some references give slightly higher values for females, possibly because they live longer, nor are there differences in terms of the different ethnic groups, despite some studies pointing to Caucasians as the most affected [2].

Patients with AMD suffer a reduced central visual acuity (VA), impaired color vision, diminished contrast sensitivity (CS), and metamorphopsia. Central vision is essential to read, drive, and recognize faces [3]. Patients with early or intermediate AMD usually have no symptoms and the VA should be good when there are no subfoveal drusen. Some patients need more light for reading or sewing [4], due to the fact that drusen can cause difficulties in darkness adaptation [5]. Patients with geographic atrophy usually have difficulties in reading, driving, and facial recognition. As far as the first affected zone is perifoveal, VA should not be affected until the disease is in an advanced stage. That often makes AMD patients feel misunderstood, because they have difficulties in reading, but they have a good VA. Visual loss is usually slow and progressive.

According to the World Health Organization (WHO), health is a state of complete physical, mental, and social well-being, and not merely the absence of disease or infirmity. The measurement of quality of life is becoming more relevant in health science, due to its evolution from a disease-based model to a patient-based model. For this reason, some questionnaires used to measure the quality of life are being introduced in the clinical practice [3].

The evolution of medicine has meant that many chronic patients live longer, meaning that it is more important to measure the quality of life [6,7,8].

AMD causes a severe decrease in quality of life. AMD patients have a higher dependency on other people, difficulties in his/her daily life, suffer more frequently depression, have a higher risk of falls and committing suicide, as well as needing residential care earlier in life [3].

A large amount of blue wavelength light can cause chromatic aberration and light dispersion, mainly in cases of damaged cones or opaque structures in the eye. The image’s quality improves when wavelengths under 450 nm are eliminated [9].

Selective cut optical filters are prescribed for people with different types of diseases, such as AMD, retinitis pigmentosa, cataracts, diabetic retinopathy, cone dystrophy, and oculo-cutaneous albinism [10]. 

Different methods have been tried to determine the best color, tint, material of the lens, or even type of frame, but there is no scientific protocol to guide the prescription of filters [11]. Nowadays, we need to be guided by our clinical observations, subjective opinions, and real-world tests of each patient [10].

In scientific literature, there is controversy in the benefits of the optical filters. Thus, results of VA, CS, glare (GL), etc., vary depending on the test used, reaching contradictory conclusions [10,12,13]. Some of the limitations of these studies are the low number of patients, the absence of AMD classification, and the use of tests that are not recognized as gold-standard [13]. 

This work aims to study whether selective cut optical filters produce an improvement in the AMD patient’s quality of life. The quality of life approach was chosen because it is an integral concept that encompasses the whole person, albeit that visual variables determine the result.

## 2. Materials and Methods

A prospective and longitudinal study, with convenience sampling, was carried out. The inclusion criteria were patients diagnosed with dry AMD that wanted to participate. It was performed in Òptica Visió and at the Ophthalmological Service in Hospital Sant Joan de Déu of Manresa, belonging to Althaia Foundation. Investigation procedures adhered to the Declaration of Helsinki and were approved by the Ethics Committee of “Fundació Unió Catalana Hospitals” with ethical code number CEI 18/86. All participants signed informed consent.

The visual variables studied were VA, CS, line difference in the high and low contrast test of Colenbrander (dlTC), and structure (STR). Early Treatment Diabetic Retinopathy Study (ETDRS) charts were used to determine VA [14] and the Pelli–Robson test to measure CS [2]. The test of Colenbrander (TC) was not used, as usual, to measure reading speed [15,16]. It was used to measure daily living activities (activities of low or medium contrast and low or medium visual acuity), because it is a mixed contrast test. It is a near vision chart in which every line has the same number of characters. The size of the letters is in metric notation (M). They vary from 10 to 0.6 M. The size of the letters is in a logarithmic progression. Spaces are proportional to the size of every letter. This test can study low (of Weber’s 20%) and high contrast VA. Finally, to determine STR, the retina was checked with a Topcon 3D OCT Maestro (Topcon, Tokyo Japan). The photographs taken by the optician were assessed by the ophthalmologists from the telemedicine company Optretina, and those from Althaia were assessed by Dr. Eduard Pedemonte. 

For the measurement of the quality of life related to vision, the National Eye Institute Vision Function Questionnaire 25 (NEI VFQ-25) was used [17,18]. It is a questionnaire designed to capture the impact of the visual problems on the physical, emotional, and social aspects of patients [19]. It is validated for AMD [20,21] and adapted to Spanish [22]. NEI VFQ-25 is composed of different subscales: general health (1 item), general vision (1 item), ocular pain (2 items), near vision (3 items), distance vision, (3 items), role limitations (2 items), mental health (4 items), role limitations (2 items), dependency (1 item), driving (2 items), color vision (1 item), and peripheral vision (1 item). Each subscale point and final score goes from 0 to 100 [23,24]. An increase of 5 points in the NEI VFQ-25 was considered as clinically relevant [25]. 

FPF (Full Protection Filters) selective cut optical filters of 450 nm, 511 nm, and 527 nm from AVS (Madrid, Spain) were used.

The procedure followed by patients was:

First visit:

- Complete visual examination based on the clinical guide of the American Optometric Association [25].
- Indoor and outdoor test with selective cut optical filters over the far vision prescription. Patients chose the most comfortable for him/her.
- Measure of CS and dlTC with and without filters.
- NEI VFQ-25.

Review after 15 days:

- NEI VFQ-25.

Three groups were defined by each visual variable. Thus, patients were divided according to their VA in group A (VA > 0.63), B (0.30 < VA ≤ 0.63) and C (VA < 0.30), based on International Classification of Diseases, Ninth Revision, Clinical Modification ICD-9-CM [26]. According to CS, subjects were divided into groups A (CS > 1.52), B (1.04 < CS < 1.52) and C (CS < 1.04), based on the Mars test [27]. While based on dlTC results, they were divided into groups A (0 ≤ TC ≤ 2), B (3 ≤ TC ≤ 5), and C (TC ≥ 6). Finally, according to STR, group A was in an early-stage, group B was in an intermediate stage, and group C was in the late-stage of AMD, according to AREDS classification [2].

The statistical analysis was performed with software R version 3.6.2 (R Foundation for Statistical Computing, Vienna, Austria 12 December 2019) and with Microsoft Excel 2016 (Microsoft, Redmond, USA). To compare NEI VFQ-25 values before and after using filters, the t Students’ test was used for the total score and each subscale. To study homogeneity in the groups, Barlett’s test was used. Welch’s ANOVA was applied following Tukey’s adjusted method of multiple comparisons, according to Herberich et al. [28]. Data were described as mean ± standard deviation and p values under 0.05 were considered statistically significant.

## 3. Results

In total, 79 subjects finished the study; 30% males and 70% females. Overall, 4% of the subjects were aged between 50 and 59, 3% between 60 and 69, 25% between 70 and 79, 51% between 80 and 89, and 17% were over 90.

Table 1 shows the number and percentage of patients who selected each filter based on the state of AMD

The results obtained in the NEI VFQ-25 for the total sample and each of the groups can be observed in Table 2. 

Table 3 shows results in each subscale of the questionnaire before and after using filters

The success of filters was defined as an increase by 5 or more points of NEI VFQ-25 values. Table 4 shows the percentage of patients in each group that showed differences equal to or higher than 5 points in the quality of life questionnaire after the use with filters in relation to the initial visit.

## 4. Discussion

The NEI VFQ-25 provides a measure of the patient’s quality of life concerning his/her vision. This allows us to work with a variable that encompasses all the usual visual variables (VA, CS, color vision, GL, and visual field), in addition to the other general health variables. It is important to highlight that the measurement of the quality of life may be used as a comparable value between different researchers. According to previous studies, a 5-point improvement in the NEI VFQ-25 is considered as clinically relevant [24]. 

This study has demonstrated how the use of filters improves the quality of life of patients with AMD. 

Nowadays, there is no standard procedure to prescribe filters. The ideal procedure according to the author’s clinical experience is testing the filters, both indoors and outdoors, to choose the best one based on the patient’s comfort. This test can be carried out in the eye professional’s office, but the best option would be providing a pair of glasses with filters for the patient, allowing her/him to test it in real conditions during her/his daily routines.

This study aimed to discover whether there was an improvement in the quality of life of AMD patients when they use filters, but a filter comparison was not made. However, we have also included results divided by the filter. In our study, patients who chose a 511 nm filter achieved the highest improvement in his/her quality of life, with an increase of 12.65 points, versus 4.76 points achieved by patients who chose 450-nm filters. It is important to highlight that filters were not assigned to patients at random, but that patients chose the most comfortable filters for themselves. This resulted in a low number of subjects using 527-nm filters, so results for this wavelength cannot be considered.

Although we expected to fit more 511-nm filters, 76% of the patients in our study chose 450-nm filters (21% of 511 nm and 3% of 527 nm). This fact could be explained by the composition of our sample (early AMD 15%, intermediate 33%, and late 53%), where the percentage of patients with early and intermediate AMD was higher than in other studies. It is also important to point out that filters are usually prescribed in the most advanced cases. The results support that a wider range of patients could benefit from using filters.

In elderly people, VA decreases. Low contrast VA starts decreasing, on average, 7 years before high contrast VA [29]. Furthermore, in patients with AMD, this loss is more pronounced [2]. 

In the Colenbrander test, a person with normal vision can have a difference of 1 or 2 lines, comparing to ETDRS VA. For a person with AMD, these differences are of the order of 5 or 6 lines, reaching even up to 10 lines. This fact is important for the early detection of the condition. AMD often starts in the perifoveal zone, so changes in contrast can be detected faster than in central VA. Changes in the first affected eye can be compensated by the healthy eye, making detection more difficult. 

In this study, through the perspective of the quality of life improvement, it has been checked that filter adaptation is more successful for patients with VA lower than 0.25 and those with late AMD. For CS lower than 1.48 and dlTC higher than 3, the success rate also increased. However, filters should always be an option, even in those groups of patients with no statistical differences, because there are also patients that can benefit from them.

Regarding subscales, the punctuation in the visual questions of NEI VFQ-25 increased in the subscale, “difficulty in far and near vision”. This could be an indicator of the improvement of vision in patients, a subject that causes great controversy among different researchers. Thus, there are many contradictory studies on whether filters improve VA, CS, color vision, or GL [10,11,12,13]. Many attempts have been made to gather conclusions from the different tests, both objective and subjective, but always without clear results. This is the first study that analyses the success of filters through a validated quality of life questionnaire with a large sample, and with patients classified by the degree of the disease. 

As a result of dealing with those patients, the great importance of GL was highlighted. Many patients reported that they had gone from a level of discomfort to a level in which they were comfortable with the amount of light. It would be of interest for future studies to add this measure to the variables to study.

No statistical inference could be performed in this study with 527-nm filters, due to the small number of cases analyzed, which is a limitation. 

## 5. Conclusions

Selective filters improve the quality of life of patients with AMD. The quality of life measurement can show comparable results between different researchers and highlight the importance of filters for eye care professionals. The success rate in the fitting of filters is better for a VA lower than 0.25 and late or advanced AMD.

## Figures and Tables

**Table 1:** Chosen filters by patients according to the state of their age-related macular degeneration (AMD).

Filter Type	Early AMD N (%)	Intermediate AMD N (%)	Advanced AMD N (%)	Total Patients N (%)
450 nm	12 (15%)	21 (27%)	27 (34%)	60 (76%)
511 nm	0 (0%)	4 (5%)	13 (16%)	17 (21%)
527 nm	0 (0%)	0 (0%)	2 (3%)	2 (3%)

AMD: Age-related macular degeneration.

**Table 2:** Results of NEI VFQ-25 global score for the total of the patients and each group.

	Final Visit (Mean ± SD)	Initial Visit (Mean ± SD)	Difference (Mean ± SD)	*p*
**Total (*n* = 79)**	79.74 ± 15.51	73.24 ± 17.26	6.49 ± 10.55	<0.001 *
**Groups According to VA**				
A: VA > 0.63 (*n* = 3 2)	90.41 ± 5.76	87.11 ± 5.55	3.30 ± 4.97	0.001 *
B: 0.30 < VA ≤ 0.63 (*n* = 31)	74.78 ± 15.33	69.26 ± 13.98	5.52 ± 10.48	0.006
C: VA < 0.25 (*n* = 16)	67.99 ± 16.48	53.23 ± 15.01	14.77 ± 14.55	0.001 *
**Groups According to CS**				
A: CS > 1.52 (*n* = 16)	89.50 ± 8.23	87.03 ± 9.56	2.47 ± 3.43	0.011 *
B: 1.04 < CS < 1.48 (*n* = 53)	79.66 ± 14.86	71.91 ± 17.18	7.75 ± 11.61	<0.001 *
C: CS < 1.00 (*n* = 10)	64.54 ± 16.46	58.28 ± 11.33	6.26 ± 11.29	0.113
**Groups According to dlTC**				
A: 0 ≤ dlTC ≤ 2 (*n* = 28)	86.79 ± 11.71	83.84 ± 14.84	2.96 ± 6.87	0.001 *
B: 3 ≤ dlTC ≤ 5 (*n* = 31)	81.36 ± 11.07	73.70 ± 13.02	7.65 ± 10.08	0.006 *
C: dlTC ≥ 6 (*n* = 20)	67.34 ± 18.98	57.70 ± 14.93	9.65 ± 14.08	0.001 *
**Groups According to STR**				
A: Early (*n* = 12)	90.76 ± 6.67	88.20 ± 6.29	2.56 ± 5.64	0.144
B: Intermediate (*n* = 25)	82.56 ± 11.75	78.41 ± 12.96	4.16 ± 8.99	0.030 *
C: Late (*n* = 42)	74.90 ± 17.34	65.90 ± 17.92	9.01 ± 11.90	<0.001 *
**Groups According to Chosen Filter**				
A: 450 nm (*n* = 60)	81.54 ± 14.49	76.78 ± 15.32	4.76 ± 8.57	<0.001 *
B: 511 nm (*n* = 17)	76.44 ± 16.99	63.79 ± 17.99	12.65 ± 13.86	0.002 *
C: 527 nm (*n* = 2)	53.63 ± 3.59	47.42 ± 24.75	6.21 ± 21.15	0.750

NEI VFQ-25: The National Eye Institute 25-Item Visual Function Questionnaire. VA: Visual Acuity, CS: Contrast Sensitivity, dlTC: line difference in the high and low contrast test Colenbrander, STR: Structure. * Subscales with statistically significant improvement.

**Table 3:** Results of NEI VFQ-25 subscales.

Subscale NEI VFQ-25	Final Visit (Mean ± SD)	Initial Visit (Mean ± SD)	Difference (Mean ±SD)	*p*
**General Health (*n* = 79)**	37.03 ± 22.60	35.13 ± 20.62	1.90 ± 25.25	0.506
**General Vision (*n* = 79)**	46.58 ± 19.67	36.46 ± 15.94	10.13 ± 18.64	<0.001 *
**Ocular Pain (*n* = 79)**	94.62 ± 8.42	90.82 ± 11.45	3.80 ± 12.07	0.006 *
**Difficulty in Activities of Near Vision (*n* = 79)**	72.20 ± 22.75	64.19 ± 26.19	8.02 ± 15.05	<0.001 *
**Difficulty in Activities of Distance Vision (*n* = 77)**	75.38 ± 22.16	60.61 ± 27.36	14.77 ± 19.62	<0.001 *
**Limitation in Social Functions Due to Vision (*n* = 79)**	81.17 ± 20.20	77.85 ± 22.37	3.32 ± 19.68	0.138
**Mental Problems Due to Vision (*n* = 79)**	77.93 ± 22.53	72.39 ± 25.99	5.54 ± 16.38	0.004 *
**Role Difficulties Due to Vision (*n* = 79)**	75.63 ± 25.86	67.25 ± 29.16	8.39 ± 20.48	<0.001 *
**Dependency of Other People (*n* = 79)**	87.76 ± 23.18	81.96 ± 27.23	5.80 ± 20.25	0.013 *
**Driving Difficulties (*n* = 15)**	79.62 ± 29.69	80.00 ± 21.32	−0.38 ± 17.80	0.936
**Color Vision Problems (*n* = 78)**	95.19 ± 12.78	91.03 ± 18.89	4.17 ± 15.30	0.019 *
**Difficulty in Peripheral Vision (*n* = 79)**	92.41 ± 16.19	91.14 ± 18.36	1.27 ± 19.57	0.567

NEI VFQ-25: The National Eye Institute 25-Item Visual Function Questionnaire. * Subscales with statistically significant improvement.

**Table 4:** Percentage of success in the use of filters in the different groups.

Group	VA *	dlTC **	CS ***	STR ^†^	Selected Filter ^‡^
**A**	31%	25%	19%	17%	40%
**B**	45%	58%	55%	32%	59%
**C**	69%	50%	30%	60%	50%

* Group A: VA > 0.63; Group B: 0.30 < VA ≤ 0.63; Group C: VA < 0.25; ** Group A: 0 ≤ dlTC ≤ 2; Group B: 3 ≤ dlTC ≤ 5; Group C: dlTC ≥ 6; *** Group A: CS > 1.52; Group B: 1.04 < CS ≤ 1.48; Group C: CS < 1.00; ^†^ Group A: Early; Group B: Intermediate; Group C: Late; ^‡^ Group A: 450 nm; Group B: 511 nm; Group C: 527 nm. VA: Visual Acuity, CS: Contrast Sensitivity, dlTC: line difference in the high and low contrast test Colenbrander, STR: Structure.
